# The relationship between fear of childbirth and birth self-efficacy in low and high-risk pregnant women

**DOI:** 10.1186/s12884-025-08354-w

**Published:** 2025-11-10

**Authors:** Özlem Çiçek, Nuran Nur Aypar Akbağ, Gamze Durmazoğlu, Çiğdem Öztürk

**Affiliations:** 1 Department of Nursing, Womens’ Health and Disease Department, Faculty of Health Science, Bakırçay University, Izmir, Turkey; 2 Department of Midwifery, Faculty of Health Science, Sinop University, Sinop, Turkey; 3 Izmir Bozyaka Training and Research Hospital, Izmir, Turkey; 4 Dokuz Eylül University Research and Application Hospital, Izmir, Turkey

**Keywords:** Fear of childbirth, Childbirth self-efficacy, Low-risky pregnancy, high-risky pregnancy

## Abstract

**Background:**

Fear of childbirth (FOC) is the most important feeling pregnant women experience about childbirth. It is also known that the fear of childbirth is affected by many factors. This study investigated the relationship between Fear of childbirth (FOC) and childbirth self-efficacy in low and high-risk pregnant women.

**Methods:**

The research was designed in descriptive and correlational type. This study among 115 low-risk and 135 high-risk pregnant women who were recorded using a purposeful sampling.

**Results:**

The total W-DEQ-A values of high-risk pregnant women are substantially higher. It was determined that both groups had a moderate FOC. The W-DEQ-A and CBSEI-32 mean scores of low-risk pregnant women were significantly related. Their mean W-DEQ-A scores were negatively correlated with outcome expectancy sub-dimension (OE) scores and positively correlated with self-efficacy expectancy sub-dimension (EE) values.

**Conclusion:**

This is the first study to examine the relationship between FOC and childbirth self-efficacy in low and high-risk pregnant women. It is recommended that health professionals evaluate all pregnant women in terms of FOC and their childbirth self-efficacy for vaginal delivery.

## Introduction

Fear of childbirth (FOC) is one of the most important emotions that pregnant women experience related to childbirth [1]. Studies show that 39–54% of women experience mild, 30–41% moderate, and 13.2–20% severe FOC [2, 3]. Recent studies conducted in Turkey have shown that pregnant women’s fear of childbirth is between 42.4% and 82.6% [4, 5] and that 21% of them experience FOC at the clinical level [6]. According to some studies, FOC is affected by many factors. These factors can be classified as socio-demographic [3, 7], obstetric [2, 8] and psychological factors [9, 10]. Some studies show that socio-demographic factors, such as education level [3, 11], employment status [3], and economic status [11, 12], and obstetric factors, such as the number of pregnancies [3, 4, 11], previous mode of birth [13], and parity, affect FOC [4, 9]. In the study by Gökçe Isbir et al. (2022), it was stated that nulliparous women experienced more FOC than multiparous women, while in the meta-analysis by Dencker et al. (2019), it was stated that the level of FOC was similar in both groups, but the reasons for fear were different [14].

Risk status during pregnancy may also affect FOC [14, 15]. The risk status in pregnancy is classified as low and high risk. A pregnancy period with a single fetus, between the ages of >18 and < 35 years, and without psychiatric disease and systemic diseases before or during pregnancy is defined as a low-risk pregnancy. Pregnancies other than low-risk are defined as high-risk pregnancies (NRC, 2013) [16]. Also, pregnancies with the mother or the developing fetus or both being at risk of complications during or after pregnancy and childbirth are defined as high-risk [17–19]. Globally, more than 20 million women have high-risk pregnancies, which kill an estimated 830 women a day. More than 99% of these deaths are more common in rural women and adolescents from developing countries [20, 21]. It is known that there are 1.3 million childbirths a year in Turkey, and 10–15% of these childbirths consist of high-risk pregnant women [22]. High-risk pregnancies are recognized as a major public health issue, and addressing the healthcare needs of high-risk pregnant women is a goal of the World Health Organization (WHO) Sustainable Development Goals 3 (SDG 3) [23].

Pregnant women’s confidence in her ability to cope with childbirth, which is expressed as childbirth self-efficacy by Lowe (2000), is a condition that is affected by the risk level of pregnancy and fear of childbirth [15, 24, 25]. It is known that pregnant women with high fear of childbirth have a decrease in their confidence in their ability to perform useful behaviors during childbirth [26] and their birth self-efficacy [25, 27]. Barut and Uçar (2018) and Schwartz et al. (2015) determined that, regardless of parity, women with high fear of childbirth had lower birth self-efficacy.

As recommended by the American Maternal-Fetal Medicine Association, nurses who are health professionals among obstetric care providers [28] play an important role in determining the levels of pregnant women’s fear of childbirth. In this case, it can be said that nurses will play a key role in increasing pregnant women’s self-confidence during childbirth by determining their fear of childbirth and increasing their birth self-efficacy. Although the number of studies on examining the relationship between fear of childbirth and birth self-efficacy is limited, there is no study examining the relationship between fear of childbirth and birth self-efficacy in low and high-risk pregnant women. Accordingly, the aim of this study is to investigate the relationship between fear of childbirth and birth self-efficacy in low- and high-risk pregnant women.

## Materials and methods

### Type of the study

The research was designed in descriptive and correlational type.

### Study setting

The data were collected in the gynecology and obstetrics clinics and polyclinics of a tertiary hospital between December 2022-December 2023.

### Sample of the study

The study sample consisted of 135 high-risk and 115 low-risk pregnant women selected using the purposeful sampling method. Inclusion criteria for low-risk pregnant women were > 18 years of age, > 24th gestational weeks, and no health problems before or during pregnancy. The criteria for high-risk pregnant women were > 24th gestational weeks, 35 < age < 18, 24.9 < BMI < 18.5, and no psychological or physiological medical diagnosis that would create maternal and neonatal risk before or during pregnancy.

### Data collection tools

Data collection tools included a personal information form, the Wijma Delivery Expectancy/Experience Questionnaire version A (W-DEQ-A) [29, 30] and Childbirth Self-Efficacy Inventory – Short Version (CBSEI-32) [24].

#### Personal information form

This form included questions about the pregnant women’s name, surname, age, education level, marital status, income status, obstetric characteristics (gestational age, obstetric history, health problems occurring before or during pregnancy), whether the pregnancy is desired by the mother and father candidates, having received childbirth preparation education, and the source of education if any.

#### The Wijma Delivery Expectancy/Experience Questionnaire Version A (W-DEQ-A)

This questionnaire was developed by Wijma, Wijma, and Zar (1998) [30] to determine the fear of childbirth and is the most frequently used tool to measure the fear of childbirth. The Turkish validity and reliability study of the scale was performed by Körükçü et al. (2012) [29], and Cronbach’s alpha coefficient was found to be 0.92. The scale, which has 33 items in total, has a six-point Likert-type structure (0 “totally”, 5 “not at all”). Scores on the scale range between 0 and 165. A high score indicates a high fear of childbirth. Körükçü et al. (2012) grouped WIJMA Delivery Expectancy/Experience Questionnaire scores under four subgroups: low-level fear of childbirth (≤ 37 points), moderate-level fear of childbirth (38–65 points), high-level fear of childbirth (66–84 points), and severe fear of childbirth (≥ 85 points). The Cronbach alpha coefficient of W-DEQ in this study was 0.91.

#### The Childbirth Self-Efficacy Inventory – Short Version (CBSEI-32)

This form was developed by Ip et al. in 2007 [31]. The Turkish validity and reliability study of the scale was conducted by Aydın et al. (2021) [32]. It consists of 28 items and two sub-dimensions, namely outcome expectancy and self-efficacy expectancy. The outcome expectancy sub-dimension (OE) consists of 12 items and the self-efficacy expectancy sub-dimension (EE) consists of 16 items. Scores on this 10-point Likert-type scale, which consists of 28 items in total, range between 28 (OE: 12, EE: 16) and 280 (OE: 120, EE: 160). Items on the OE sub-dimension are scored between 1 “not at all useful” and 10 “very useful” and those on the EE are scored between 1 “completely sure” and 10 “not sure at all”. High scores on the scale indicate high outcome expectancy and self-efficacy expectancy of pregnant women about childbirth. Cronbach’s alpha values of CBSEI-32 and OE and EE sub-dimensions are 0.82, 0.82, and 0.90, respectively. In our study, Cronbach’s alpha values of CBSEI-32 and OE and EE sub-dimensions were found as 0.57, 0.91, and 0.81, respectively.

### Data analysis

Research data were analyzed on the SPSS 22.00 software package. The characteristics of the pregnant women were analyzed using frequency and percentage values. The relationship between the mean scores of low-risk and high-risk pregnant women in the sample on the W-DEQ-A and CBSEI-32 and their sub-dimensions was analyzed using Spearman’s correlation analysis. The analysis of the effects of personal and obstetric characteristics on the total W-DEQ-A and CBSEI-32 and sub-dimension scores was conducted using a t-test for parametric data and the Mann-Whitney U test for non-parametric data. The relationship between parity and CBSEI-32 and W-DEQ-A scores by risk groups was evaluated using Spearman’s correlation analysis, and the mean scores on the CBSEI-32, OE, and EE were analyzed using the Kruskal-Wallis test. Statistical significance was taken as *p* < 0.05.

## Results

Of the pregnant women participating in the study, 46% had a low-risk pregnancy and 54% had a high-risk pregnancy. The mean age of the low-risk pregnant women was 27.37 ± 4.67 years, 35.7% of them were high school graduates, the mean gestational age was 28.72 ± 8.87 weeks, and 45.2% were multiparous. The mean age of high-risk pregnant women was 29.64 ± 5.85 years, 26.7% of them were high school graduates, the mean gestational age was 27.13 ± 9.32 weeks, and 50.4% were multiparous (Table 1).

**Table 1:** Socio-demographic and obstetric characteristics of low-risk and high-risk pregnant women

	Low-risk pregnant women (*n* = 115)	High-risk pregnant women (*n* = 135)
Age$\:(\overline{\mathbf{x}}\pm\:\mathbf{S}\mathbf{D}$)	27.37$\:\pm\:$4.67	29.64$\:\pm\:$5.85
Level of education (*n*, %)		
Elementary school	20 (17.4)	19 (14.1)
Middle school	25 (21.7)	31 (23)
High school	41 (35.7)	36 (26.7)
University	29 (25.2)	49 (36.3)
Gestational age$\:(\overline{\mathbf{x}}\pm\:\mathbf{S}\mathbf{D}$)	28.72$\:\pm\:$8.87	27.13$\:\pm\:$9.32
Parity (*n*, %)		
Nulliparous	63 (54.8)	67 (49.6)
Multiparous	52 (45.2)	68 (50.4)

$\:\overline{\text{x}}$ Mean, *SD* Standard Deviation

When high-risk pregnant women were classified in terms of risk, it was determined that most of the risks occurred during pregnancy. Diabetes Mellitus (7.4%) had the highest incidence among the systemic diseases existing before pregnancy. Of the high-risk pregnant women who participated in the study, 24.4% had conceived over the age of 35. Also, 22.2% of pregnant women had a history of abortion or a diagnosis of abortus imminent. The health problems that posed a risk during pregnancy included bleeding (30.4%), development of contraction (28.1%), problems related to blood pressure (12.6%), thyroid disease (8.1%), and gestational diabetes mellitus (8.1%).

The mean scores of low-risk and high-risk pregnant women on the W-DEQ-A were 56.12 ± 22.24 and 59.45 ± 24.46, respectively. It was determined that both groups had a moderate fear of childbirth. There was significant difference between the mean scores of low-risk and high-risk pregnant women on the fear of childbirth (X^2^ = 107.547, *p* = 0.036). However, there was no significant difference between the mean scores of low-risk and high-risk pregnant women on the self-efficacy at birth (X^2^ = 83.755, *p* = 0.205) (Table 2). The mean scores of low-risk and high-risk pregnant women on the total CBSEI-32 were 203.30 ± 19.46 and 201.94 ± 20.05, respectively. Regarding the sub-dimension scores, the mean scores of low-risk and high-risk pregnant women were 134.06 ± 20.93 and 131.50 ± 25.70 on the OE sub-dimension and 69.24 ± 17.26 and 70.43 ± 21.57 on the EE sub-dimension, respectively. No statistically significant difference was found between CBSEI-32 and sub-dimensions by risk group (Table 2).

**Table 2:** Mean scores of low-risk and high-risk pregnant women on the W-DEQ-A and CBSEI-32

	Low-risk pregnant women (*n* = 115)			High-risk pregnant women (*n* = 135)			X ^2^	*p*
	*n*, %	$\:\overline{\mathbf{x}}\pm\:\mathbf{S}\mathbf{D}$	min-max	*n*, %	$\:\overline{\mathbf{x}}\pm\:\mathbf{S}\mathbf{D}$	min-max		
W-DEQ-A		56.12 ± 22.24	6–109		59.45 ± 24.46	10–118	107.547	0.036*
≤ 37 (those with a low level of fear of childbirth)	28, %24.3	27.21 ± 8.13	6–37	29, %21.5	25.65 ± 8.33	10–37	26.991	0.171
38–65 (those with a moderate level of fear of childbirth)	50, %43.5	53.96 ± 6.73	38–65	51, %37.8	54.05 ± 8.40	38–65	32.452	0.216
66–84 (those with a severe level of fear of childbirth)	25, %21.7	73.44 ± 5.36	67–84	36, %26.7	73.33 ± 5.57	66–84	26.340	0.092
≥ 85 (those with a clinical level of fear of childbirth)	12, %10.4	96.50 ± 9.68	85–109	19, %14.1	99.26 ± 10.56	86–118	21.025	0.101
CBSEI-32		203.30 ± 19.46	157–257		201.94 ± 20.05	149–264	83.755	0.205
OE		134.06 ± 20.93	47–160		131.50 ± 25.70	31–160	61.713	0.627
EE		69.24 ± 17.26	43–114		70.43 ± 21.57	36–134	69.246	0.435

*W-DEQ-A* Wijma Delivery Expectancy/Experience Questionnaire version A, *CBSEI-32* Childbirth Self-Efficacy Inventory – Short Version, *OE* Outcome Expectancy, EE: Efficacy Expectancy,$\:\overline{\text{x}}$ Mean, *SD* Standard Deviation, *X*^2^ Chi-square test, *p < 0.05

The significant relationship was found between low-risk pregnant women’s mean scores on the W-DEQ-A and the CBSEI-32 (*r*=−0.192, *p* = 0.039). Their mean scores on the W-DEQ-A had a significant negative correlation with mean OE scores (*r*=−0.443, *p* = 0.000) and a significant positive correlation with mean EE scores (*r* = 0.378, *p* = 0.000). It was determined that there was no significant relationship between the mean self-efficacy scores according to the fear of childbirth classification (Table 3).

**Table 3:** Relationship between mean W-DEQ-A and CBSEI-32 scores

		CBSEI-32		OE		EE	
		r**	*p*	r	*p*	r	*p*
Low-risk pregnant women (*n*=115)	W-DEQ-A Classification						
	Mean W-DEQ-A score	−0.192	0.039*	−0.443	0.000*	0.378	0.000*
	≤37 (low-level fear of childbirth)	0.283	0.145	0.144	0.464	0.153	0.438
	38–65 (moderate-level fear of childbirth)	−0.024	0.867	0.022	0.882	0.139	0.337
	66–84 (severe-level fear of childbirth)	0.068	0.748	0.378	0.063	−0.215	0.303
	≥85 (clinical-level fear of childbirth)	−0.011	0.972	−0.117	0.718	0.266	0.404
High-risk pregnant women (*n*=135)	W-DEQ-A Classification						
	Mean W-DEQ-A score	−0.114	0.188	−0.450	0.000*	0.420	0.000*
	≤37 (low-level fear of childbirth)	0.218	0.255	−0.135	0.467	0.347	0.065
	38–65 (moderate-level fear of childbirth)	−0.188	0.187	−0.266	0.060	0.133	0.352
	66–84 (severe-level fear of childbirth)	−0.032	0.852	−0.132	0.442	0.171	0.320
	≥85 (clinical-level fear of childbirth)	0.276	0.254	0.107	0.661	0.201	0.410

*W-DEQ-A* Wijma Delivery Expectancy/Experience Questionnaire version A, *CBSEI-32* Childbirth Self-Efficacy Inventory – Short Version, *OE* Outcome Expectancy, *EE* Efficacy Expectancy, *p<0.05,**r=Spearman’s correlation test

There was no significant relationship between high-risk pregnant women’s mean scores on the W-DEQ-A and the CBSEI-32 (*r*=−0.114, *p* = 0.188). On the other hand, their mean scores on the W-DEQ-A had a significant negative correlation with mean OE scores (*r*=−0.450, *p* = 0.000) and a significant positive correlation with mean EE scores (*r* = 0.420, *p* = 0.000) (Table 3).

## Discussion

### Fear of childbirth in high- and low-risk pregnant women

Fear of childbirth is a common problem experienced by many pregnant women. In our study, it was determined that both high-risk and low-risk pregnant women had a similarly moderate fear of childbirth. As a result of his studies and observations, Grantly Dick-Read realized that when there was no fear at birth, there was no pain and defined the fear-tension-pain triangle in the 1920s. According to Dick Read’s philosophy, fear of the unknown causes tension, and tension causes pain [33]. Pain experienced during childbirth causes an increase in fear, making women more sensitive to pain [34].

Although the pregnant women in our study experienced a moderate level of fear of childbirth, the mean fear of childbirth score of high-risk pregnant women was found to be higher than that of low-risk ones. When the literature was examined, it was determined that the fear of childbirth in low-risk pregnant women was moderate, similar to our study [3, 4]. Similar to the results of this study, other studies found that high-risk pregnant women had a higher level of fear of childbirth than low-risk ones [35, 36]. Hess et al. (2004) determined that the most important factor causing fear of childbirth in pregnant women was the fear of losing their baby [37]. The higher fear of loss experienced by women with high-risk pregnancies can be explained by the increased fear of loss. The reason why the rates of severe- (26.7%) and clinical-level fear of childbirth (14.1%) were higher in high-risk pregnant women who participated in our study compared to the low-risk group (22.2% and 6.7%, respectively) may be because they experienced fear of loss. For this reason, it is thought that the fear of pregnant women who are worried about the continuity of the pregnancy process may increase due to the uncertainties about the timing of the birth and how it will take place.

Determination of the factors affecting the fear of childbirth and birth self-efficacy in the prenatal period helps to identify pregnant women who may experience severe fear of childbirth. Identification of expectant mothers who have a high level of fear of childbirth during pregnancy will enable health professionals to support and guide them and make appropriate interventions to improve their birth self-efficacy by providing them with psychological support.

In this study, it was determined that socio-demographic and obstetric characteristics of pregnant women had no effect on the fear of childbirth. In another study conducted in Turkey, it was found that there was a significant negative correlation between fear of childbirth and age and that as the education level of pregnant women increased, their fear of childbirth decreased. Similar to this study, gestational age or parity was not found to be associated with fear of childbirth [38].

Some studies have shown that factors, such as parity [39, 40], gestational week [41] planned pregnancy [42], and history of abortion [15], affect the fear of childbirth. Ilska et al. (2021) [43] investigated the relationship between obstetric factors and fear of childbirth with 359 women who were in the third trimester of pregnancy. In the study, no significant difference was found between parity and fear of childbirth. In the study by Akın Utku with a total of 300 pregnant women, including 150 non-risky primigravidas and 150 multigravidas, it was determined that mean scores on the total WIJMA were higher in primigravidas (73.16 ± 9.38) than in multigravidas (71.55 ± 6.37) (p˂0.05) [39]. However, no significant difference was found between the groups in terms of the severity of the fear of childbirth. In this study, it was determined that the parity status of low- and high-risk pregnant women did not affect their mean fear of childbirth scores. According to the study of Onchonga et al., 13.8% of low-risk nulliparous pregnant women experienced severe fear of childbirth, while this rate was 8% in low-risk multiparous pregnant women [8].

On the other hand, İsbir et al. (2022) and Kabukcu et al. (2019) found that multiparous women experienced higher levels of fear of childbirth than nulliparous women [2, 4]. Studies show that the fear of childbirth in nulliparous women is due to not knowing what they will encounter during childbirth, while the fear of childbirth in multiparous women arises from past birth experiences [4]. In a study conducted in Australia, socio-demographic, obstetric, and psychological factors associated with the fear of childbirth in nulliparous and multiparous women were examined. In the study, a correlation was found between fear of childbirth, parity, and the mode of last childbirth. It was determined that the fear levels of first-time mothers were higher than those of women who had given birth before. It was found that a normal previous birth experience was protective against the fear of childbirth and that women who had given birth that required interventions had more fear of childbirth [40].

Mohamamdirizi et al. (2018) also evaluated the fear of childbirth in high-risk and low-risk pregnant women [36]. As in our study, no difference was found between high and low-risk pregnant women in terms of fear of childbirth, but the mean fear of childbirth score of pregnant women in the high-risk group was found to be higher than the score of those in the low-risk group.

### Birth self-efficacy in high- and low-risk pregnant women

One of the most important factors in the development of behaviors to cope with the pain and fear of childbirth is birth self-efficacy. The more developed the coping ability of the pregnant woman is, the less she feels the birth pain [27]. Women with high birth self-efficacy tend to internalize, master, and perform certain tasks expected of them during childbirth [44]. According to self-efficacy theory, people with high coping efficacy adopt strategies and courses of action designed to transform negative situations into more positive ones. The stronger the sense of competency is, the braver people are in taking on problematic situations that cause stress and the more successful they are in shaping these conditions as they wish [45, 46]. As an expected result, it was found that the high-risk pregnant women in this study had lower self-efficacy levels than the low-risk ones. However, it was determined that there was no statistically significant difference between the groups. In the study by Sánchez-Cunqueiro, Comeche, and Docampo (2018) [47], the relationship between self-efficacy expectancy, use of coping strategies during childbirth, and postpartum satisfaction was evaluated. Only low-risk pregnant women were included in this study. A positive correlation was found between scores on the self-efficacy expectancy and coping skills during childbirth. It was found that women with higher self-efficacy scores used coping strategies during childbirth and had a more positive evaluation of the birth experience.

Our study results indicated that the age and educational status of high-risk and low-risk pregnant women had no effect on their birth self-efficacy, of the low-risk pregnant women, multiparas had lower birth self-efficacy than nulliparas, and that the difference was statistically significant. An Australian study on the examination of the relationship between birth self-efficacy and socio-demographic, obstetric, and psychological factors indicated that similar to our study, there was no relationship between birth self-efficacy and age and educational status in nulliparous or multiparous women. However, contrary to our study, the birth self-efficacy of multiparas was higher than that of the nulliparas [25].

In the study by Ölçer, Bakır and Oskay, the perception of self-efficacy was evaluated only in high-risk pregnancies (2016) [48]. It was determined that self-efficacy increased with age, and the self-efficacy of pregnant women who had their first pregnancy, had not given birth before, or had no living children was low. In a study conducted in China to evaluate the birth self-efficacy of pregnant women and the factors affecting it, similar to our results, it was determined that multiparas had lower birth self-efficacy perceptions than nulliparas and that there was no significant difference between mean birth self-efficacy scores and gestational age [49].

In this study, birth self-efficacy perceptions of high-risk pregnant women with a younger gestational age were found to be higher than the perceptions of those with an older gestational age. In the study of Pan et al. (2019) [50] in which they examined the results of the mindfulness-based birth and parenting program, it was observed that similar to the results of this study, the birth self-efficacy scores of pregnant women decreased in the 36th gestational week.

### The relationship between birth self-efficacy and fear of childbirth

According to our research results, there was no significant relationship between the fear of childbirth and self-efficacy levels of low- and high-risk pregnant women. However, in both groups, it was revealed that the fear of childbirth had a negative relationship with the outcome expectancy sub-dimension of the birth self-efficacy and a positive relationship with the self-efficacy expectancy sub-dimension. It was determined that as the fear of childbirth decreased, birth self-efficacy increased. Barut and Uçar (2018) conducted a descriptive study in a training and research hospital to determine the relationship between the perception of birth self-efficacy and fear of childbirth. As a result of the study, it was determined that as pregnant women’s birth self-efficacy increased, their fear of childbirth decreased [27]. Similarly, in a study conducted with low-risk pregnant women in China, it was found that fear of childbirth and birth self-efficacy were closely related. Stronger fear of childbirth resulted in lower birth self-efficacy and lower birth self-efficacy resulted in increased fear of childbirth. For this reason, it is recommended to evaluate pregnant women in terms of fear of childbirth symptoms and do timely interventions [51].

## Conclusion and recommendations

This is the first study to examine the relationship between fear of childbirth and birth self-efficacy in low-and high-risk pregnant women. As a result of the study, it was determined that the parity status of low-risk pregnant women and the gestational age of high-risk pregnant women affected birth self-efficacy. In addition, it was determined that both groups had a moderate fear of childbirth. The mean W-DEQ-A score had a negative relationship with the mean outcome expectancy score and a positive significant relationship with the mean self-efficacy expectancy sub-dimension score.

In line with this information, it is thought that women with a high level of birth self-efficacy will have a high expectancy of coping with childbirth and giving a vaginal birth. The fear of childbirth and self-efficacy perceptions of pregnant women who are preparing for childbirth should be increased with family-hospital collaboration. Prenatal education affects the adaptation to the pregnancy process, prepares the pregnant woman for birth, eliminates the unknown, and therefore increases birth self-efficacy [44, 46, 47]. In this context, it is important to carry out applications to reduce the fear of childbirth and increase the self-efficacy perceptions of pregnant women in prenatal education.

Several methods can play a role in increasing self-efficacy. One of these methods is pregnancy yoga. In addition to traditional yoga elements, it is known that safe yoga training includes techniques for coping with birth pain and training content in which concerns, experiences, and stories can be shared, and it reduces the fear of childbirth and increases women’s self-efficacy perceptions [52]. For these reasons, determination of the fear of childbirth and self-efficacy levels of pregnant women during childbirth preparation education is important in creating the content of education programs.

## Data Availability

The datasets used and/or analyzed during the current study are available from the corresponding author on reasonable request.
